# Editorial: Using Noise to Characterize Vision

**DOI:** 10.3389/fpsyg.2015.01707

**Published:** 2015-11-16

**Authors:** Remy Allard, Jocelyn Faubert, Denis G. Pelli

**Affiliations:** ^1^Aging in Vision and Action Laboratory, Université Pierre et Marie CurieParis, France; ^2^Visual Psychophysics and Perception Laboratory, Universiteì de MontréalMontréal, QC, Canada; ^3^Department of Psychology and Center for Neural Science, New York UniversityNew York, NY, USA

**Keywords:** noise, equivalent input noise, linear amplifier model, perceptual template model, noise image classification, bandpass noise, contrast jitter, phase noise

Auditory noise is a sound, a random variation in air pressure. More generally, random “noise” can be introduced into any stimulus, including a visual display. Noise added to the stimulus can probe the computations underlying perception of the stimulus. With power and precision, the noise, by restricting the information available, places fundamental constraints on attainable performance and processing strategy. WWII research on radar led to mathematical theorems about detectability of signals in noise, i.e., Signal Detection Theory (Peterson et al., 1954), which allow human performance to be expressed on an absolute scale of efficiency, 0–100% (Tanner and Birdsall, 1958; Pelli and Farell, 1999). Auditory noise revealed the channels of hearing in studies at Bell Labs that characterized how telephone line noise limits perception of speech (Fletcher, 1953). Studies of visual effects of photographic, x-ray, and video noise (reviewed in Pelli, 1981) led to pioneering work with artificially injected noise by Rose (1957), Stromeyer and Julesz (1972), and Solomon and Pelli (1994). Added visual noise has been widely used to characterize the computations underlying various visual tasks (e.g., detection, discrimination, letter and face recognition, search, averaging, selective attention, perceptual learning) in various populations (e.g., older adults, amblyopes, migrainers, dyslexic children). Different kinds of noise probe different aspects of the computation. For instance, spectrally filtered noise is used to determine the frequencies relevant to a given visual task (e.g., letter identification, Solomon and Pelli, 1994). Noise masking of one attribute (e.g., in luminance, color, or texture) can reveal whether another attribute is processed separately (e.g., Gegenfurtner and Kiper, 1992; Allard and Faubert, 2007, 2008). Noise image classification can reveal the visual features the observer uses to perform a visual task (e.g., Eckstein and Ahumada, 2002). Noise is also often used to characterize what limits sensitivity, such as internal noise (Pelli, 1981; Pelli and Farell, 1999; Lu and Dosher, 2008). This Research Topic issue explores effective ways to use noise to probe visual function.

“Noise” in perception experiments generally means unpredictable variation in some aspect of the stimulus. Typically, the stimulus consists of a luminance signal plus an unpredictable noise. Less often, another parameter of the signal, e.g., orientation, varies unpredictably (e.g., Dakin, 1999; Solomon, 2010; Allard and Cavanagh, 2012). Added noise is often *white*: A random sample, independent and identically distributed, is added to each pixel's luminance. The extent of the noise is restricted, or “localized,” by a window in space and time. The spatiotemporal spectrum of the noise can be restricted by bandpass filtering to a range of orientation and frequency. Added noise that varies across space is sometimes called “pixel noise.” Most of the studies in this Research Topic issue added noise to the signal; two studies randomly jittered parameters of the signal.

In this Research Topic issue, Jeon et al. (2014) added localized white noise to investigate developmental changes in orientation discrimination through childhood. Interpreting their data using the Perceptual Template Model (Lu and Dosher, 2008), to see how the model parameters change with age, they find that increasing age reduces internal additive noise, reduces internal multiplicative noise, and improves external noise exclusion. Using a similar noise paradigm, Chou et al. (2014) find that localized attention facilitated contrast detection due to signal enhancement, whereas object-based attention facilitated detection due to external noise exclusion. Letter identification is mediated by an octave-wide spatial frequency channel (Solomon and Pelli, 1994). Young and Smithson (2014) use spatially bandpass noise to reveal the letter identification channel in the presence of optical distortions, and find changes in the central spatial frequency of the letter-identification channel. Hall et al. (2014) find that adding white noise increased the center spatial frequency of the letter-identification channel for large but not small letters. Using pixel noises with different spectral profiles, Abbey and Eckstein (2014) find that performance approaches that of the mathematical ideal in a free-localization task (i.e., high spatial uncertainty), but is much lower in a fixed-location task (i.e., low spatial uncertainty), indicating that the human detection strategy is well-adapted to free-localization tasks. Gold (2014) use pixel noise to investigate the visual information used by the observer during a size-contrast illusion. By correlating the observers' classification decision with each pixel of the noise stimuli, they find that the spatial region used to estimate the size of the target is influenced by the size of surrounding irrelevant elements. Taylor et al. (2014) use pixel noise both as a target and a mask. The target noise is bandpass-filtered in orientation and spatial frequency, whereas the mask is white noise. They find that information used to detect the target is more optimal in the orientation domain than in the frequency domain, suggesting that observers can adjust the bandwidth of their channels in orientation, but not in spatial frequency.

Several studies examine how visual processing is affected by the extent and bandwidth of applied noise. Baker and Vilidaite (2014) provide EEG evidence that white noise masks have a suppressive gain control effect on neural responses to grating stimuli. Happily, Allard and Faubert (2014b) note that suppressive gain control would not affect threshold measurements in white noise. Studying motion perception, Allard and Faubert (2014a) find similar orientation and direction thresholds with and without temporally extended noise, but greater direction thresholds in temporally localized noise. This shows that the processing strategy underlying motion perception depends on the noise duration. Consistent with previous studies on contrast sensitivity (Allard and Cavanagh, 2011; Allard et al., 2013), they conclude that to measure equivalent input noise of motion processing, noise should be temporally extended (e.g., displayed continually).

Two studies randomly jittered a signal parameter. In an electrophysiological study, Németh et al. (2014) use *phase noise*, produced by randomizing phases in the Fourier domain, making the stimulus unrecognizable without affecting its spectral energy. Thus, sensitivity to phase noise suggests involvement in recognition. They find that phase-noise amplifies the P1 response to cars in the right hemisphere, but not in the left hemisphere, and that, conversely, phase-noise amplifies the P1 response to faces in the left hemisphere, but not in the right hemisphere. Lidestam et al. (2014) evaluate the effect of informational and energetic auditory noise on visual speechreading. They found that only informational auditory noise (i.e., four-talker babble) interfered with speechreading, which suggests that phonological processing is also involved in speechreading.

In sum, this Research Topic issue shows several ways to use diverse kinds of noise to probe visual processing.

## Author contributions

RA wrote the editorial, which was substantially improved by DP and proof read by JF.

### Conflict of interest statement

The authors declare that the research was conducted in the absence of any commercial or financial relationships that could be construed as a potential conflict of interest.
